# 1,8-Bis(4-chloro­benzo­yl)-7-meth­oxy­naphthalen-2-ol ethanol monosolvate

**DOI:** 10.1107/S1600536810024074

**Published:** 2010-06-26

**Authors:** Ryosuke Mitsui, Atsushi Nagasawa, Keiichi Noguchi, Akiko Okamoto, Noriyuki Yonezawa

**Affiliations:** a Department of Organic and Polymer Materials Chemistry, Tokyo University of Agriculture & Technology, 2-24-16 Naka-machi, Koganei, Tokyo 184-8588, Japan; b Instrumentation Analysis Center, Tokyo University of Agriculture & Technology, 2-24-16 Naka-machi, Koganei, Tokyo 184-8588, Japan

## Abstract

In the title compound, C_25_H_16_Cl_2_O_4_·C_2_H_6_O, the two 4-chloro­benzoyl groups are in *syn* orientations with respect to the naphthalene ring system and are approximately parallel to each other: the dihedral angle between the benzene rings is 11.43 (16)°. The conformation around each of the carbonyl C—(C=O)—C groups forms a larger angle to the plane of the naphthalene ring system than that to the benzene ring; the angles of the C=O bond vector with the naphthalene ring system and the benzene ring are 55.4 (3) *versus* 13.5 (3)° and 52.2 (3) *versus* 17.9 (3)°. An intra­molecular O—H⋯O=C hydrogen bond generates a six-membered ring. In the crystal structure, inter­molecular O—H⋯O hydrogen bonds including the ethanol solvent mol­ecule are observed. A C—H⋯O inter­action also occurs. The ethyl group of the ethanol mol­ecule is disordered over two positions with site occupancies of 0.63 and 0.37. The crystal studied was an inversion twin.

## Related literature

For the structures of closely related compounds, see: Mitsui *et al.* (2008 ▶); Nakaema *et al.* (2007 ▶).
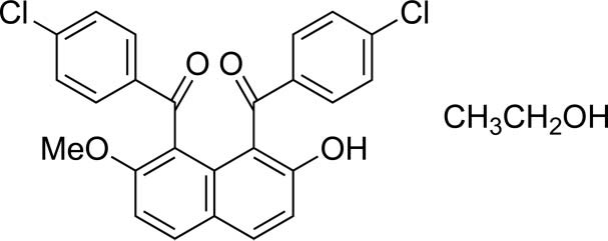


## Experimental

### 

#### Crystal data

C_25_H_16_Cl_2_O_4_·C_2_H_6_O
*M* *_r_* = 497.35
Tetragonal, 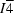

*a* = 25.2992 (5) Å
*c* = 7.3068 (2) Å
*V* = 4676.71 (18) Å^3^
*Z* = 8
Cu *K*α radiation
μ = 2.81 mm^−1^
*T* = 193 K
0.60 × 0.10 × 0.10 mm

#### Data collection

Rigaku R-AXIS RAPID diffractometer
Absorption correction: numerical (*NUMABS*; Higashi, 1999 ▶) *T*_min_ = 0.283, *T*_max_ = 0.766
43771 measured reflections
4272 independent reflections
3676 reflections with *I* > 2σ(*I*)
*R*_int_ = 0.059

#### Refinement

*R*[*F* ^2^ > 2σ(*F* ^2^)] = 0.050
*wR*(*F* ^2^) = 0.146
*S* = 1.11
4272 reflections
322 parameters
50 restraints
H atoms treated by a mixture of independent and constrained refinement
Δρ_max_ = 0.41 e Å^−3^
Δρ_min_ = −0.24 e Å^−3^
Absolute structure: Flack (1983 ▶), 1951 Friedel pairs
Flack parameter: 0.425 (17)

### 

Data collection: *PROCESS-AUTO* (Rigaku, 1998 ▶); cell refinement: *PROCESS-AUTO*; data reduction: *CrystalStructure* (Rigaku/MSC, 2004 ▶); program(s) used to solve structure: *SIR2004* (Burla *et al.*, 2005 ▶); program(s) used to refine structure: *SHELXL97* (Sheldrick, 2008 ▶); molecular graphics: *ORTEPIII* (Burnett & Johnson, 1996 ▶); software used to prepare material for publication: *SHELXL97*.

## Supplementary Material

Crystal structure: contains datablocks I, global. DOI: 10.1107/S1600536810024074/is2562sup1.cif

Structure factors: contains datablocks I. DOI: 10.1107/S1600536810024074/is2562Isup2.hkl

Additional supplementary materials:  crystallographic information; 3D view; checkCIF report

## Figures and Tables

**Table 1:** Hydrogen-bond geometry (Å, °)

*D*—H⋯*A*	*D*—H	H⋯*A*	*D*⋯*A*	*D*—H⋯*A*
O4—H4*O*⋯O2	0.82	2.21	2.900 (4)	141
O4—H4*O*⋯O5^i^	0.82	2.32	2.919 (5)	131
O5—H5*O*⋯O4^ii^	0.90 (1)	1.83 (1)	2.636 (4)	147 (2)
C21—H21⋯O2^iii^	0.95	2.58	3.374 (5)	141

Symmetry codes: (i) 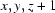; (ii) 
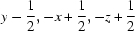
; (iii) 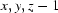.

**Fig. 1.**
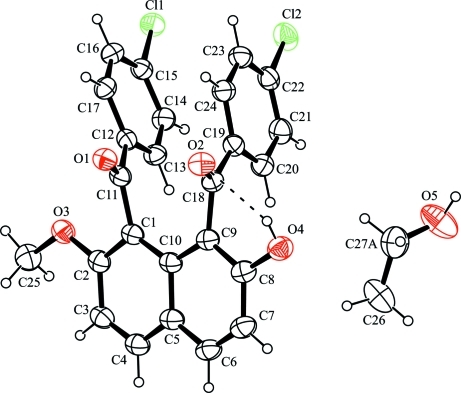
The asymmetric unit of compound (II), showing 30% probability displacement ellipsoids. Only major parts of the disordered atoms are shown. The intramolecular O—H···O═C hydrogen bond is shown as a dashed line.

**Fig. 2.**
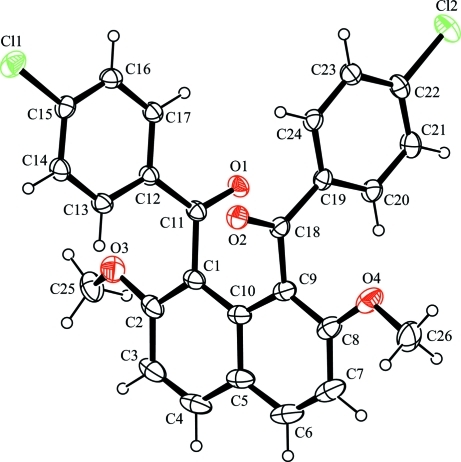
The asymmetric unit of compound (I), showing 30% probability displacement ellipsoids.

**Fig. 3.**
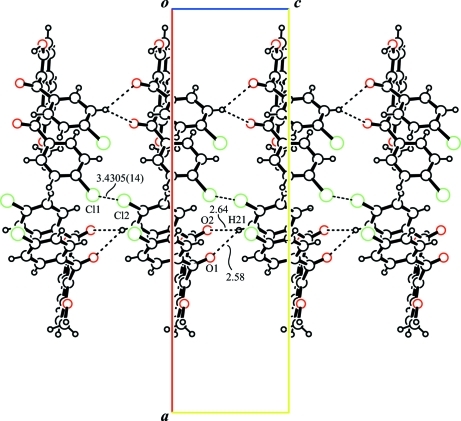
Partial crystal packing diagram of compound (II), viewed down the b axis. Intermolecular C—H···O hydrogen bonds and Cl···Cl and H···O interactions are shown as dashed lines.

**Fig. 4.**
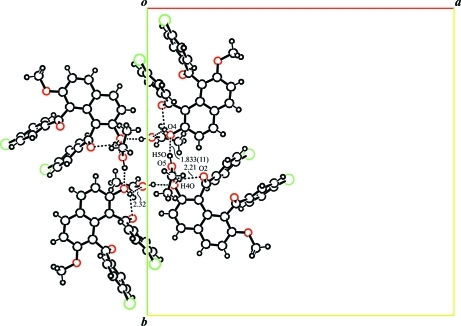
Partial crystal packing diagram of compound (II), viewed down the c axis. The intramolecular O—H···O═C hydrogen bond and the intermolecular O—H···O hydrogen bonds are shown as dashed lines.

## supplementary crystallographic information

### Comment

Recently, we reported the crystal structures of aroylated 2,7-dimethoxynaphthalenes, 1,8-bis(4-chlorobenzoyl)-2,7-dimethoxynaphthalene, (I) (Nakaema *et al.*, 2007) and (4-chlorophenyl)(2-hydroxy-7-methoxynaphthalen-1-yl)methanone (Mitsui *et al.*, 2008). As a part of our ongoing studies on the synthesis and crystal structure analysis of aroylated naphthalene derivatives, we prepared and analysed the structure of crystal of 1,8-bis(4-chlorobenzoyl)-2-hydroxy-7-methoxynaphthalene, (II). The title compound was prepared by electrophilic aromatic aroylation reaction of (4-chlorophenyl)(2-hydroxy-7-methoxynaphthalen-1-yl)methanone with 4-chlorobenzoyl chloride.

An *ORTEPIII* (Burnett & Johnson, 1996) plot of (II) is shown in Fig. 1. In analogous aroylated naphthalenes, for example compound (I) shown in Fig. 2, has two methoxy groups on the naphthalene ring, and the two 4-chlorobenzoyl groups that are in an *anti* orientation against the naphthalene ring system. In contrast, compound (II) has one hydroxy group instead of methoxy group, and the conformation of 4-chlorobenzoyl groups in compound (II) are in a *syn* orientation. The two benzene rings are nearly parallel, the dihedral angle between the benzene rings are 11.43 (16)°. The conformation around the central carbonyl C—(C═O)—C group is such that the C═O bond vector forms a larger angle to the plane of the naphthalene ring system [C1/C2/C3/C4/C5/C10 ring and C5/C6/C7/C8/C9/C10 ring] than that to the plane of the benzene ring [C12/C13/C14/C15/C16/C17 ring and C19/C20/C21/C22/C23/C24 ring], *viz.* 55.4 (3)° *versus* 13.5 (3)° and 52.2 (3)° *versus* 17.9 (3)°, respectively. The intramolecular O—H···O═C hydrogen bond generates a six-membered ring (Figs. 1 and 4, Table 1).

In the crystal structure, Cl1 and Cl2 interact with each other [Cl1···Cl2 = 3.4305 (14) Å] and the 4-chlorobenzoyl groups interact with the carbonyl groups (H21···O1 = 2.64 Å, H21···O2 = 2.58 Å) along the *c* axis (Fig. 3 and Table 1). The hydroxy groups in the compound (II) and the ethanol molecule act as a hydrogen-bond donor [H5O···O4 = 1.833 (11) Å, H4O···O5 = 2.32 Å], and these intermolecular O—H···O hydrogen bonds connecting the compound (II) and the ethanol molecule contribute to the stabilization of the molecular conformation and crystal structure (Fig. 4 and Table 1).

### Experimental

To a solution of (4-chlorophenyl)(2-hydroxy-7-methoxynaphthalen-1-yl)methanone (62 mg, 0.20 mmol) in dichloromethane (0.1 ml) was added 4-chlorobenzoyl chloride (77 mg, 0.44 mmol) and titanium tetrachloride (250 mg, 1.32 mmol). The reaction mixture was heated at reflux for 3 h, then poured into H_2_O (10 ml), and the aqueous layer was extracted with CHCl_3_ (3 × 10 ml). The combined organic layers were washed with saturated NaHCO_3_ (3 × 30 ml) and brine (3 × 30 ml), and dried over MgSO_4_ overnight. The solvent was removed *in vacuo* and the crude material was purified by column chromatography (silica gel, 1:1 EtOAc:hexane) to give the title compound (yield 73 mg, 81%). Single crystals suitable for X-ray diffraction analysis were obtained from EtOH as yellow platelet (m.p. 505.5–506.5 K).

Spectroscopic Data: ^1^H NMR (300 MHz, CDCl_3_) δ 9.34 (s, 1H), 7.94–7.86 (m, 2H), 7.33 (d, 2H), 7.20–7.10 (m, 6H), 6.91 (d, 2H), 3.60 (s, 3H); ^13^C NMR (75 MHz, CDCl_3_) δ 196.8, 195.1, 159.5, 157.6, 139.7, 138.4, 136.8, 136.3, 135.0, 133.7, 132.6, 131.9, 130.5, 128.5, 127.7, 124.6, 121.3, 117.3, 110.6, 56.0; IR (KBr): 1643, 1612, 1587, 1510, 1278, 1089, 831; HRMS (*m/z*): [*M* + H]^+^ calcd for C_25_H_17_O_4_Cl_2_, 451.0504; found, 451.0520. Anal. Calcd for C_25_H_16_O_4_Cl_2_: C 66.53, H 3.57. Found: C 66.31, H 3.76.

### Refinement

All H atom were found in difference maps and were subsequently refined as riding atoms, with C—H = 0.95 (aromatic), 0.98 (methyl), 0.99 (methylene) Å and O—H = 0.83 Å, and with *U*_iso_ (H) = 1.2*U*_eq_ (C, O). The ethyl chain of the ethanol molecule is disordered over two positions with occupancies of 0.63 and 0.37. In the ethanol molecule, C—C, C—O and O—H distances were restrained to 1.50 (1), 1.40 (1) and 0.90 (1) Å [5 restraints with the *DFIX* command in *SHELXL97* (Sheldrick, 2008)]. C27A—H5O and C27B—H5O distances were restrained to 1.90 (1) Å (2 restraints with *DANG* command in SHLEXL97). Rigid bond restraints were applied to the *U*_ij_ values of the methoxy group (C25 and O3) and the ethanol molecule (C26, C27A, C27B, O5) (7 restraints with the *DELU* command in *SHELXL97*). Further restraints were used to generate similar *U^ij^* values for the atoms of ethanol molecule (36 restraints with the *SIMU* command in *SHELXL97*).

### Crystal data

**Table:**

C_25_H_16_Cl_2_O_4_·C_2_H_6_O	*D*_x_ = 1.413 Mg m^−^^3^
*M**_r_* = 497.35	Melting point = 505.5–506.5 K
Tetragonal, *I*4	Cu *K*α radiation, λ = 1.54187 Å
Hall symbol: I -4	Cell parameters from 34431 reflections
*a* = 25.2992 (5) Å	θ = 3.5–68.2°
*c* = 7.3068 (2) Å	µ = 2.81 mm^−^^1^
*V* = 4676.71 (18) Å^3^	*T* = 193 K
*Z* = 8	Platelet, yellow
*F*(000) = 2064	0.60 × 0.10 × 0.10 mm

### Data collection

**Table:**

Rigaku R-AXIS RAPID diffractometer	4272 independent reflections
Radiation source: rotating anode	3676 reflections with *I* > 2σ(*I*)
graphite	*R*_int_ = 0.059
Detector resolution: 10.00 pixels mm^-1^	θ_max_ = 68.2°, θ_min_ = 3.5°
ω scans	*h* = −30→30
Absorption correction: numerical (*NUMABS*; Higashi, 1999)	*k* = −29→30
*T*_min_ = 0.283, *T*_max_ = 0.766	*l* = −8→8
43771 measured reflections	

### Refinement

**Table:**

Refinement on *F*^2^	Hydrogen site location: inferred from neighbouring sites
Least-squares matrix: full	H atoms treated by a mixture of independent and constrained refinement
*R*[*F*^2^ > 2σ(*F*^2^)] = 0.050	*w* = 1/[σ^2^(*F*_o_^2^) + (0.0883*P*)^2^] where *P* = (*F*_o_^2^ + 2*F*_c_^2^)/3
*wR*(*F*^2^) = 0.146	(Δ/σ)_max_ = 0.001
*S* = 1.11	Δρ_max_ = 0.41 e Å^−^^3^
4272 reflections	Δρ_min_ = −0.24 e Å^−^^3^
322 parameters	Extinction correction: *SHELXL97* (Sheldrick, 2008), Fc^*^=kFc[1+0.001xFc^2^λ^3^/sin(2θ)]^-1/4^
50 restraints	Extinction coefficient: 0.00147 (14)
Primary atom site location: structure-invariant direct methods	Absolute structure: Flack (1983), 1951 Friedel pairs
Secondary atom site location: difference Fourier map	Flack parameter: 0.425 (17)

### Special details

**Table:**

Geometry. All e.s.d.'s (except the e.s.d. in the dihedral angle between two l.s. planes) are estimated using the full covariance matrix. The cell e.s.d.'s are taken into account individually in the estimation of e.s.d.'s in distances, angles and torsion angles; correlations between e.s.d.'s in cell parameters are only used when they are defined by crystal symmetry. An approximate (isotropic) treatment of cell e.s.d.'s is used for estimating e.s.d.'s involving l.s. planes.
Refinement. Refinement of *F*^2^ against ALL reflections. The weighted *R*-factor *wR* and goodness of fit *S* are based on *F*^2^, conventional *R*-factors *R* are based on *F*, with *F* set to zero for negative *F*^2^. The threshold expression of *F*^2^ > σ(*F*^2^) is used only for calculating *R*-factors(gt) *etc*. and is not relevant to the choice of reflections for refinement. *R*-factors based on *F*^2^ are statistically about twice as large as those based on *F*, and *R*- factors based on ALL data will be even larger.

### Fractional atomic coordinates and isotropic or equivalent isotropic displacement parameters (Å^2^)

**Table:**

	*x*	*y*	*z*	*U*_iso_*/*U*_eq_	Occ. (<1)
Cl1	0.05719 (3)	0.03898 (3)	0.17865 (14)	0.0621 (3)	
Cl2	−0.02496 (4)	0.16783 (4)	0.08841 (16)	0.0770 (3)	
O1	0.11592 (9)	0.21618 (9)	0.8263 (4)	0.0551 (6)	
O2	0.04808 (10)	0.31611 (10)	0.8034 (4)	0.0612 (6)	
O3	0.23047 (8)	0.17133 (9)	0.6041 (4)	0.0598 (6)	
O4	0.07574 (10)	0.41241 (9)	0.6105 (4)	0.0678 (7)	
H4O	0.0566	0.3955	0.6795	0.081*	
C1	0.17824 (11)	0.24765 (13)	0.6109 (5)	0.0479 (7)	
C2	0.22785 (13)	0.22437 (13)	0.5843 (5)	0.0522 (8)	
C3	0.27296 (13)	0.25544 (15)	0.5449 (5)	0.0566 (9)	
H3	0.3063	0.2393	0.5240	0.068*	
C4	0.26750 (14)	0.30881 (15)	0.5375 (5)	0.0584 (9)	
H4	0.2979	0.3298	0.5153	0.070*	
C5	0.21886 (14)	0.33392 (14)	0.5614 (5)	0.0545 (8)	
C6	0.21545 (15)	0.39011 (14)	0.5502 (5)	0.0612 (9)	
H6	0.2467	0.4099	0.5287	0.073*	
C7	0.16923 (16)	0.41585 (14)	0.5690 (6)	0.0645 (10)	
H7	0.1681	0.4533	0.5609	0.077*	
C8	0.12273 (14)	0.38691 (13)	0.6008 (5)	0.0560 (8)	
C9	0.12359 (12)	0.33224 (12)	0.6194 (5)	0.0506 (8)	
C10	0.17206 (12)	0.30356 (13)	0.5983 (5)	0.0489 (7)	
C11	0.13535 (12)	0.21062 (13)	0.6754 (5)	0.0488 (7)	
C12	0.11815 (12)	0.16695 (12)	0.5514 (5)	0.0464 (7)	
C13	0.13067 (13)	0.16708 (13)	0.3672 (5)	0.0521 (8)	
H13	0.1527	0.1942	0.3194	0.063*	
C14	0.11142 (14)	0.12794 (14)	0.2511 (5)	0.0548 (8)	
H14	0.1190	0.1288	0.1238	0.066*	
C15	0.08117 (12)	0.08796 (12)	0.3244 (5)	0.0501 (7)	
C16	0.06922 (13)	0.08626 (13)	0.5068 (5)	0.0535 (8)	
H16	0.0488	0.0579	0.5545	0.064*	
C17	0.08686 (12)	0.12576 (13)	0.6207 (5)	0.0527 (8)	
H17	0.0778	0.1252	0.7469	0.063*	
C18	0.07059 (13)	0.30794 (12)	0.6591 (5)	0.0513 (8)	
C19	0.04529 (13)	0.27497 (13)	0.5116 (5)	0.0510 (8)	
C20	0.06054 (14)	0.27961 (14)	0.3330 (6)	0.0572 (8)	
H20	0.0868	0.3047	0.3000	0.069*	
C21	0.03752 (15)	0.24741 (15)	0.1975 (6)	0.0625 (9)	
H21	0.0478	0.2503	0.0729	0.075*	
C22	−0.00009 (14)	0.21190 (14)	0.2508 (6)	0.0588 (9)	
C23	−0.01677 (14)	0.20716 (14)	0.4298 (6)	0.0610 (9)	
H23	−0.0430	0.1820	0.4622	0.073*	
C24	0.00536 (13)	0.23966 (13)	0.5612 (6)	0.0560 (8)	
H24	−0.0065	0.2380	0.6844	0.067*	
C25	0.28018 (15)	0.14558 (17)	0.5787 (7)	0.0720 (11)	
H25A	0.2756	0.1073	0.5930	0.086*	
H25B	0.3054	0.1585	0.6700	0.086*	
H25C	0.2936	0.1532	0.4556	0.086*	
C26	0.10138 (18)	0.4338 (3)	0.0813 (9)	0.1041 (17)	
H26A	0.1190	0.4317	0.2004	0.125*	0.63
H26B	0.1041	0.4700	0.0339	0.125*	0.63
H26C	0.1183	0.4093	−0.0045	0.125*	0.63
H26D	0.1226	0.4626	0.1330	0.125*	0.37
H26E	0.1193	0.4193	−0.0264	0.125*	0.37
H26F	0.0969	0.4059	0.1731	0.125*	0.37
C27A	0.0461 (3)	0.4197 (4)	0.1019 (11)	0.092 (2)	0.63
H27A	0.0445	0.3839	0.1557	0.111*	0.63
H27B	0.0302	0.4443	0.1919	0.111*	0.63
C27B	0.0485 (4)	0.4546 (5)	0.028 (2)	0.092 (4)	0.37
H27C	0.0313	0.4689	0.1394	0.110*	0.37
H27D	0.0539	0.4845	−0.0571	0.110*	0.37
O5	0.01506 (14)	0.42004 (17)	−0.0507 (6)	0.1179 (14)	
H5O	−0.0194 (3)	0.4263 (11)	−0.026 (3)	0.141*	

### Atomic displacement parameters (Å^2^)

**Table:**

	*U*^11^	*U*^22^	*U*^33^	*U*^12^	*U*^13^	*U*^23^
Cl1	0.0672 (5)	0.0567 (5)	0.0624 (6)	−0.0070 (4)	−0.0069 (4)	−0.0074 (4)
Cl2	0.0848 (6)	0.0637 (5)	0.0826 (7)	0.0000 (4)	−0.0310 (6)	−0.0117 (5)
O1	0.0530 (12)	0.0687 (14)	0.0436 (13)	−0.0058 (10)	0.0053 (11)	−0.0048 (11)
O2	0.0612 (14)	0.0643 (14)	0.0580 (16)	−0.0030 (11)	0.0122 (13)	−0.0105 (13)
O3	0.0514 (12)	0.0651 (14)	0.0630 (16)	0.0046 (10)	0.0009 (12)	−0.0015 (12)
O4	0.0671 (15)	0.0577 (14)	0.0787 (19)	−0.0015 (11)	0.0040 (14)	0.0011 (13)
C1	0.0464 (15)	0.0612 (18)	0.0363 (17)	−0.0059 (13)	0.0009 (13)	−0.0056 (14)
C2	0.0526 (18)	0.063 (2)	0.0414 (18)	−0.0029 (14)	−0.0019 (15)	−0.0039 (16)
C3	0.0462 (17)	0.078 (2)	0.0453 (19)	−0.0041 (15)	0.0027 (15)	−0.0040 (17)
C4	0.055 (2)	0.077 (2)	0.0428 (19)	−0.0147 (17)	−0.0010 (16)	−0.0027 (17)
C5	0.0551 (18)	0.066 (2)	0.0423 (18)	−0.0132 (15)	0.0018 (15)	−0.0058 (16)
C6	0.069 (2)	0.063 (2)	0.052 (2)	−0.0227 (17)	0.0005 (18)	−0.0029 (17)
C7	0.074 (2)	0.0544 (19)	0.065 (2)	−0.0153 (17)	0.002 (2)	−0.0025 (18)
C8	0.0626 (19)	0.0550 (18)	0.051 (2)	−0.0080 (15)	0.0049 (17)	−0.0070 (16)
C9	0.0531 (17)	0.0539 (17)	0.0447 (18)	−0.0067 (13)	0.0020 (15)	−0.0040 (15)
C10	0.0501 (17)	0.0591 (18)	0.0374 (17)	−0.0100 (13)	−0.0018 (14)	−0.0068 (14)
C11	0.0437 (15)	0.0582 (18)	0.0446 (18)	−0.0023 (13)	0.0004 (15)	−0.0016 (16)
C12	0.0424 (15)	0.0536 (17)	0.0431 (18)	0.0012 (12)	−0.0031 (14)	−0.0002 (14)
C13	0.0530 (17)	0.0567 (18)	0.047 (2)	−0.0071 (13)	0.0036 (15)	0.0008 (15)
C14	0.0595 (19)	0.062 (2)	0.0432 (18)	−0.0040 (16)	0.0029 (15)	−0.0015 (16)
C15	0.0479 (16)	0.0499 (16)	0.0527 (19)	−0.0020 (13)	−0.0034 (15)	−0.0039 (16)
C16	0.0545 (18)	0.0499 (18)	0.056 (2)	−0.0046 (14)	0.0048 (15)	0.0036 (15)
C17	0.0553 (17)	0.0524 (17)	0.050 (2)	0.0004 (14)	0.0052 (15)	0.0027 (15)
C18	0.0519 (17)	0.0506 (17)	0.051 (2)	−0.0009 (13)	0.0021 (16)	−0.0004 (15)
C19	0.0485 (17)	0.0518 (18)	0.053 (2)	0.0015 (14)	−0.0018 (15)	−0.0007 (15)
C20	0.0587 (19)	0.0565 (19)	0.056 (2)	−0.0049 (15)	−0.0040 (17)	0.0011 (17)
C21	0.071 (2)	0.066 (2)	0.050 (2)	0.0013 (17)	−0.0074 (18)	−0.0045 (18)
C22	0.058 (2)	0.0518 (19)	0.066 (2)	0.0023 (15)	−0.0163 (17)	−0.0054 (17)
C23	0.0553 (19)	0.0515 (18)	0.076 (3)	−0.0047 (14)	−0.0056 (19)	−0.0023 (18)
C24	0.0534 (18)	0.0540 (18)	0.061 (2)	−0.0039 (14)	0.0014 (16)	0.0005 (17)
C25	0.068 (2)	0.080 (3)	0.067 (3)	0.0053 (18)	0.006 (2)	−0.002 (2)
C26	0.078 (3)	0.140 (5)	0.095 (4)	0.015 (3)	−0.018 (3)	−0.023 (4)
C27A	0.104 (5)	0.093 (5)	0.080 (6)	0.009 (4)	−0.002 (4)	−0.002 (4)
C27B	0.080 (7)	0.095 (9)	0.102 (11)	−0.004 (6)	0.001 (7)	−0.012 (8)
O5	0.0704 (19)	0.158 (3)	0.125 (4)	0.016 (2)	−0.007 (2)	−0.054 (3)

### Geometric parameters (Å, °)

**Table:**

Cl1—C15	1.743 (3)	C16—C17	1.375 (5)
Cl2—C22	1.746 (4)	C16—H16	0.9500
O1—C11	1.215 (4)	C17—H17	0.9500
O2—C18	1.216 (4)	C18—C19	1.506 (5)
O3—C2	1.351 (4)	C19—C20	1.366 (6)
O3—C25	1.428 (4)	C19—C24	1.396 (5)
O4—C8	1.354 (4)	C20—C21	1.408 (5)
O4—H4O	0.8200	C20—H20	0.9500
C1—C2	1.400 (5)	C21—C22	1.365 (5)
C1—C10	1.426 (5)	C21—H21	0.9500
C1—C11	1.509 (4)	C22—C23	1.379 (6)
C2—C3	1.415 (5)	C23—C24	1.383 (5)
C3—C4	1.358 (5)	C23—H23	0.9500
C3—H3	0.9500	C24—H24	0.9500
C4—C5	1.396 (5)	C25—H25A	0.9800
C4—H4	0.9500	C25—H25B	0.9800
C5—C6	1.427 (5)	C25—H25C	0.9800
C5—C10	1.437 (4)	C26—C27A	1.451 (7)
C6—C7	1.345 (6)	C26—C27B	1.488 (8)
C6—H6	0.9500	C26—H26A	0.9800
C7—C8	1.405 (5)	C26—H26B	0.9800
C7—H7	0.9500	C26—H26C	0.9800
C8—C9	1.390 (5)	C26—H26D	0.9800
C9—C10	1.433 (5)	C26—H26E	0.9800
C9—C18	1.503 (4)	C26—H26F	0.9800
C11—C12	1.494 (5)	C27A—O5	1.364 (7)
C12—C13	1.382 (5)	C27A—H27A	0.9900
C12—C17	1.403 (4)	C27A—H27B	0.9900
C13—C14	1.392 (5)	C27B—O5	1.347 (8)
C13—H13	0.9500	C27B—H27C	0.9900
C14—C15	1.377 (5)	C27B—H27D	0.9900
C14—H14	0.9500	O5—H5O	0.904 (10)
C15—C16	1.367 (5)		
			
C2—O3—C25	118.8 (3)	O2—C18—C9	121.0 (3)
C8—O4—H4O	107.7	O2—C18—C19	121.1 (3)
C2—C1—C10	120.4 (3)	C9—C18—C19	117.9 (3)
C2—C1—C11	115.3 (3)	C20—C19—C24	120.5 (3)
C10—C1—C11	123.9 (3)	C20—C19—C18	121.1 (3)
O3—C2—C1	116.5 (3)	C24—C19—C18	118.4 (3)
O3—C2—C3	122.3 (3)	C19—C20—C21	120.3 (3)
C1—C2—C3	121.2 (3)	C19—C20—H20	119.8
C4—C3—C2	118.6 (3)	C21—C20—H20	119.8
C4—C3—H3	120.7	C22—C21—C20	117.9 (4)
C2—C3—H3	120.7	C22—C21—H21	121.0
C3—C4—C5	122.5 (3)	C20—C21—H21	121.0
C3—C4—H4	118.8	C21—C22—C23	122.7 (3)
C5—C4—H4	118.8	C21—C22—Cl2	118.5 (3)
C4—C5—C6	120.0 (3)	C23—C22—Cl2	118.6 (3)
C4—C5—C10	120.4 (3)	C22—C23—C24	118.9 (3)
C6—C5—C10	119.6 (3)	C22—C23—H23	120.6
C7—C6—C5	121.9 (3)	C24—C23—H23	120.6
C7—C6—H6	119.0	C23—C24—C19	119.5 (4)
C5—C6—H6	119.0	C23—C24—H24	120.2
C6—C7—C8	119.5 (3)	C19—C24—H24	120.2
C6—C7—H7	120.2	O3—C25—H25A	109.5
C8—C7—H7	120.2	O3—C25—H25B	109.5
O4—C8—C9	118.9 (3)	H25A—C25—H25B	109.5
O4—C8—C7	119.7 (3)	O3—C25—H25C	109.5
C9—C8—C7	121.4 (3)	H25A—C25—H25C	109.5
C8—C9—C10	120.4 (3)	H25B—C25—H25C	109.5
C8—C9—C18	114.3 (3)	C27A—C26—H26A	109.5
C10—C9—C18	125.2 (3)	C27A—C26—H26B	109.5
C1—C10—C9	126.1 (3)	H26A—C26—H26B	109.5
C1—C10—C5	116.9 (3)	C27A—C26—H26C	109.5
C9—C10—C5	117.0 (3)	H26A—C26—H26C	109.5
O1—C11—C12	121.2 (3)	H26B—C26—H26C	109.5
O1—C11—C1	120.2 (3)	C27B—C26—H26D	109.3
C12—C11—C1	118.6 (3)	C27B—C26—H26E	109.9
C13—C12—C17	118.8 (3)	H26D—C26—H26E	109.5
C13—C12—C11	121.5 (3)	C27B—C26—H26F	109.2
C17—C12—C11	119.6 (3)	H26D—C26—H26F	109.5
C12—C13—C14	120.8 (3)	H26E—C26—H26F	109.5
C12—C13—H13	119.6	O5—C27A—C26	117.9 (6)
C14—C13—H13	119.6	O5—C27A—H27A	107.8
C15—C14—C13	118.7 (3)	C26—C27A—H27A	107.8
C15—C14—H14	120.7	O5—C27A—H27B	107.8
C13—C14—H14	120.7	C26—C27A—H27B	107.8
C16—C15—C14	121.7 (3)	H27A—C27A—H27B	107.2
C16—C15—Cl1	119.8 (3)	O5—C27B—C26	116.6 (8)
C14—C15—Cl1	118.5 (3)	O5—C27B—H27C	108.1
C15—C16—C17	119.7 (3)	C26—C27B—H27C	108.1
C15—C16—H16	120.2	O5—C27B—H27D	108.1
C17—C16—H16	120.2	C26—C27B—H27D	108.1
C16—C17—C12	120.3 (3)	H27C—C27B—H27D	107.3
C16—C17—H17	119.9	C27B—O5—H5O	114.2 (14)
C12—C17—H17	119.9	C27A—O5—H5O	113.4 (14)
			
C25—O3—C2—C1	−179.9 (3)	O1—C11—C12—C13	165.1 (3)
C25—O3—C2—C3	−2.0 (5)	C1—C11—C12—C13	−15.8 (5)
C10—C1—C2—O3	178.1 (3)	O1—C11—C12—C17	−12.4 (5)
C11—C1—C2—O3	5.4 (5)	C1—C11—C12—C17	166.7 (3)
C10—C1—C2—C3	0.1 (5)	C17—C12—C13—C14	1.8 (5)
C11—C1—C2—C3	−172.5 (3)	C11—C12—C13—C14	−175.7 (3)
O3—C2—C3—C4	−176.3 (3)	C12—C13—C14—C15	−2.3 (5)
C1—C2—C3—C4	1.6 (5)	C13—C14—C15—C16	0.8 (5)
C2—C3—C4—C5	−2.2 (5)	C13—C14—C15—Cl1	−179.9 (3)
C3—C4—C5—C6	−179.3 (4)	C14—C15—C16—C17	1.1 (5)
C3—C4—C5—C10	1.1 (5)	Cl1—C15—C16—C17	−178.1 (2)
C4—C5—C6—C7	178.8 (4)	C15—C16—C17—C12	−1.6 (5)
C10—C5—C6—C7	−1.6 (6)	C13—C12—C17—C16	0.2 (5)
C5—C6—C7—C8	0.0 (6)	C11—C12—C17—C16	177.8 (3)
C6—C7—C8—O4	−176.6 (4)	C8—C9—C18—O2	−67.1 (4)
C6—C7—C8—C9	2.2 (6)	C10—C9—C18—O2	113.8 (4)
O4—C8—C9—C10	176.0 (3)	C8—C9—C18—C19	110.5 (4)
C7—C8—C9—C10	−2.8 (6)	C10—C9—C18—C19	−68.6 (5)
O4—C8—C9—C18	−3.2 (5)	O2—C18—C19—C20	157.9 (4)
C7—C8—C9—C18	178.1 (4)	C9—C18—C19—C20	−19.7 (5)
C2—C1—C10—C9	178.9 (3)	O2—C18—C19—C24	−21.6 (5)
C11—C1—C10—C9	−9.1 (5)	C9—C18—C19—C24	160.9 (3)
C2—C1—C10—C5	−1.2 (5)	C24—C19—C20—C21	−2.3 (5)
C11—C1—C10—C5	170.8 (3)	C18—C19—C20—C21	178.2 (3)
C8—C9—C10—C1	−178.9 (3)	C19—C20—C21—C22	0.0 (5)
C18—C9—C10—C1	0.2 (6)	C20—C21—C22—C23	1.2 (6)
C8—C9—C10—C5	1.2 (5)	C20—C21—C22—Cl2	−174.6 (3)
C18—C9—C10—C5	−179.8 (3)	C21—C22—C23—C24	0.0 (6)
C4—C5—C10—C1	0.6 (5)	Cl2—C22—C23—C24	175.8 (3)
C6—C5—C10—C1	−179.0 (3)	C22—C23—C24—C19	−2.4 (5)
C4—C5—C10—C9	−179.4 (3)	C20—C19—C24—C23	3.5 (5)
C6—C5—C10—C9	0.9 (5)	C18—C19—C24—C23	−177.0 (3)
C2—C1—C11—O1	114.4 (4)	C27B—C26—C27A—O5	49.3 (9)
C10—C1—C11—O1	−58.0 (5)	C27A—C26—C27B—O5	−49.3 (9)
C2—C1—C11—C12	−64.7 (4)	C26—C27B—O5—C27A	48.9 (9)
C10—C1—C11—C12	122.9 (3)	C26—C27A—O5—C27B	−51.4 (9)

### Hydrogen-bond geometry (Å, °)

**Table:**

*D*—H···*A*	*D*—H	H···*A*	*D*···*A*	*D*—H···*A*
O4—H4O···O2	0.82	2.21	2.900 (4)	141
O4—H4O···O5^i^	0.82	2.32	2.919 (5)	131
O5—H5O···O4^ii^	0.90 (1)	1.83 (1)	2.636 (4)	147 (2)
C21—H21···O2^iii^	0.95	2.58	3.374 (5)	141

Symmetry codes: (i) *x*, *y*, *z*+1; (ii) *y*−1/2, −*x*+1/2, −*z*+1/2; (iii) *x*, *y*, *z*−1.
